# Effects of High Intensity Interval vs. Endurance Training on Cardiac Parameters in Ischemia/Reperfusion of Male Rats: Focus on Oxidative Stress

**DOI:** 10.3389/fphys.2021.534127

**Published:** 2021-02-22

**Authors:** Marina Rankovic, Vladimir Jakovljevic, Jovana Bradic, Biljana Jakovljevic, Vladimir Zivkovic, Ivan Srejovic, Sergey Bolevich, Isidora Milosavljevic, Jovana Jeremic, Marko Ravic, Olja Mijanovic, Tamara Nikolic Turnic, Nevena Jeremic

**Affiliations:** ^1^Department of Pharmacy, Faculty of Medical Sciences, University of Kragujevac, Kragujevac, Serbia; ^2^Department of Physiology, Faculty of Medical Sciences, University of Kragujevac, Kragujevac, Serbia; ^3^Department of Human Pathology, First Moscow State Medical University IM Sechenov, Moscow, Russia; ^4^Department of Health Care, High Medical College of Professional Studies in Belgrade, Belgrade, Serbia; ^5^Institute of Regenerative Medicine, First Moscow State Medical University IM Sechenov, Moscow, Russia

**Keywords:** endurance training, high intensity interval training, ischemic reperfusion injury, rats, heart

## Introduction

Cardiovascular disease (CVD), such as heart ischemic or coronary artery disease, continues to be one of the major causes of death worldwide. Risk factors that have been considered to contribute to CVD development involve genetic features in combination with age and factors such as dyslipidemia, atherosclerosis, and hypertension. Additionally, the severity of these diseases is strongly affected by excess caloric intake and a lack of physical activity in a patient's lifestyle (Feng et al., 2019). Exercise as a category of physical activity is frequently regarded as an effective treatment to improve cardiovascular function, and it is also hypothesized that exercise training may be cardioprotective against ischemia–reperfusion (I/R) injury (Ghahremani et al., 2018). Although numerous data support the fact that regular physical activity improves heart function (Green and Smith, 2018; Pinckard et al., 2019), the optimal duration, frequency, and intensity of exercise (Serrano-Ostáriz et al., 2011) as well as the exact mechanism of this training-induced beneficial effect remains a matter of debate.

It is well-established that exercise training is beneficial not only for cardiovascular but also for non-cardiovascular systems such as endocrine, immune system, and osteomuscular apparatus. Moreover, physical exercise possesses the capability of enhancing the antioxidant defense system, thus decreasing the level of lipid peroxidation both in adults and in aged individuals (Meijers and de Boer, 2017; Bouzid et al., 2018; Simioni et al., 2018). High-volume endurance training (ET), characterized by repeated sessions of continuous moderate intensity exercise, usually involves walking or cycling for 30–60 min to reach 40–80% peak oxygen uptake (VO_2_max) (Fletcher et al., 2013). On the other hand, endurance training induces numerous physiological and biochemical adaptations that improve the oxidative capacity of skeletal muscles, modify the energy source used during effort episodes, and increase aerobic capacity (de Lade et al., 2018; Kelly et al., 2018). Furthermore, positive adaptations of this type of exercise on the cardiovascular system are also based on physiological remodeling with elevated O_2_ consumption and improvement of cardiac contractile function (Rivas-Estany et al., 2013). Nevertheless, ET offers not only protection against regional ischemia; it also prevents decreasing of heart oxygen consumption subsequently after ischemia/reperfusion (I/R) injury (Li et al., 2014). While this type of exercise offers significant training adaptations, it requires a large time commitment. Therefore, researchers have been encouraged to begin studying new approaches to exercise such as high-intensity interval training (HIIT) with possibly the same positive effects on the cardiovascular system as ET.

HIIT refers to repeated sessions of brief intermittent high-intensity exercise, between 80 and 100% of peak heart rate, interspersed with recovery periods or light exercise (Costa et al., 2018). It is often performed with maximal effort or intensity (i.e., to achieve ≥90% VO_2_max) and proposed as an alternative regimen to traditional ET to improve cardiovascular health in individuals with CVD (Xie et al., 2017). During the past decades, great scientific attention has been focused on investigating the impact of HIIT due to its potential superior ability to improve cardiorespiratory fitness for a lesser weekly time commitment in comparison to ET. Literature data suggest that HIIT improves flow-mediated dilation in individuals with impaired vascular function related to obesity, hypertension, metabolic syndrome, type 2 diabetes, and coronary artery disease to a greater magnitude than ET (Costa et al., 2018; Hannan et al., 2018). Also, this type of training improves mitochondrial biogenesis, insulin sensitivity (Kessler et al., 2012) and glucose regulation (Gibala et al., 2012), HDL cholesterol, blood pressure (Kessler et al., 2012), and deep abdominal adiposity (Boutcher, 2011) more than ET, which is of great importance for patients with CVD.

To have a more complete picture of the effects of HIIT in cardioprotection, we also created a pathological heart model. Animal studies have shown that physical exercise including both HIIT and ET confers cardioprotection against all levels of I/R-induced injury (Calvert, 2011; Frasier et al., 2011). Exercise-induced cardiovascular adaptations are considered to be intensity-dependent; however, there is still a knowledge gap regarding the exact and precise intensity of training leading to optimal cardiac effects, and the underlying mechanisms are still lacking. Taking into consideration that many people avoid regular exercise due to lack of time, the main question of our research was to investigate if high-intensity interval exercise could provide favorable effects on the cardiovascular system as well as usual moderate activity. Moreover, the purpose of this study was to reveal potential differences between two exercise regimens with emphasis on changes in redox status.

## Materials and Methods

### Animal Model

The present study was carried out on 36 male Wistar albino rats (11 ± 1 week, bodyweight 180 ± 20 g). They were housed under controlled environmental conditions, at room temperature (22 ± 2°C) with an established photo-period of 12 h light/day in the Institute of Cardiovascular Physiology, Faculty of Medical Sciences, University of Kragujevac. For research purposes, all experimental animals were obtained from the Military Medical Academy, Belgrade, Serbia. The rats had free access to food and tap water—*ad libitum*. Animals in this study were healthy and had not been treated with any drugs or supplements. Rats were randomly divided into three groups (12 animals per group): a sedentary control group (CTRL), an endurance training group (ETr), and a high-intensity interval training group (HIITr). Animals from the second group received an endurance training treatment before *in vitro* ischemia/reperfusion injury on the Langendorff apparatus (ETr), and the animals from the third group received a high-intensity interval training treatment prior to *in vitro* ischemia/reperfusion injury on the Langendorff apparatus (HIITr). The experimental protocol was approved by the Faculty of Medical Sciences Ethics Committee for the welfare of experimental animals, University of Kragujevac, number 01-13340/1 and by the Ministry of Agriculture, Forestry and Water Management, Authority for Veterinary of Serbia number 323-07-04422/2017-05 and followed the Guidelines for the Care and Use of Laboratory Animals.

### Exercise Protocols

Two different modes of intensive exercise training were used on rats: traditional endurance training (ETr) and high-intensity interval training (HIITr). ETr and HIITr programs were progressive in nature and programmed after the animals completed an initial graded treadmill exercise test to assess specific work capacity. The exercise protocols included treadmill running at 0% inclination (Rahimi et al., 2015). The rats on exercise ETr protocol ran on a treadmill for 4 weeks, 5 days weekly, with 1 week before the adaptation period (8 m/min speed for 30 min/day). After each week of training protocol, speed was gradually increased from 10 m/min in the second week to 15 m/min in the fifth week (Table 1). The exercise HIITr group ran on treadmill for 4 weeks, 5 days weekly with 1 week before the adaptation period (7 m/min speed for 15 min/day). Before the actual experiment, they just had 5 min of warmup at 8 m/min as the habituation period to the treadmill. Program details for HIITr protocol is presented in Table 2.

**Table 1:** Protocol for endurance training for rats (ETr).

Weeks/Days^*^^,^^**^	**Mo**	**Tu**	**We**	**Th**	**Fr**
1	8 m/min for 30 min				
2	10 m/min for 1 h	10 m/min for 1 h	10 m/min for 1 h	10 m/min for 1 h	10 m/min for 1 h
3	12 m/min for 1 h	12 m/min for 1 h	12 m/min for 1 h	12 m/min for 1 h	12 m/min for 1 h
4	13 m/min for 1 h	13 m/min for 1 h	13 m/min for 1 h	13 m/min for 1 h	13 m/min for 1 h
5	15 m/min for 1 h	15 m/min for 1 h	15 m/min for 1 h	15 m/min for 1 h	15 m/min for 1 h

* *3-min rest/100 m*.

** *5-min warmup at 8 m/min prior to each training session*.

**Table 2:** Protocol for high intensity interval training for rats (HIITr).

Weeks/Days^*^^,^^**^	**Mo**	**Tu**	**We**	**Th**	**Fr**
1	7 m/min for 15 min				
2	5 sprints × 45 m/min for 30 s	5 sprints × 46 m/min for 30 s	5 sprints × 47 m/min for 30 s	5 sprints × 48 m/min for 30 s	5 sprints × 49 m/min for 30 s
3	5 sprints × 50 m/min for 30 s	5 sprints × 50 m/min for 40 s	5 sprints × 50 m/min for 45 s	5 sprints × 50 m/min for 55 s	5 sprints × 50 m/min for 60 s
4	5 sprints × 51 m/min for 60 s	5 sprints × 52 m/min for 60 s	5 sprints × 53 m/min for 60 s	5 sprints × 54 m/min for 60 s	5 sprints × 55 m/min for 60 s
5	5 sprints × 55 m/min for 65 s	5 sprints × 55 m/min for 70 s	5 sprints × 55 m/min for 75 s	5 sprints × 55 m/min for 80 s	5 sprints × 55 m/min for 90 s

* *2-min rest after each sprint*.

** *5-min warmup at 8 m/min prior to each training session*.

### Preparation of Isolated Rat Heart

The hearts of male Wistar albino rats were excised and perfused on a Langendorff apparatus (Experimetria Ltd, 1062 Budapest, Hungary) following treatments. Thirty-six hours after the last physical capacity test, after a short-term ketamine/xylazine narcosis, the animals were killed by cervical dislocation (Schedule 1 of the Animals/Scientific Procedures, Act 1986, UK) and premedicated with heparin as an anticoagulant (Stanojevic et al., 2016). After emergency thoracotomy and rapid cardiac arrest by superfusion with ice-cold isotonic saline, the aortas were rapidly excised, cannulated, and retrogradely perfused under a constant perfusion pressure (CPP).

The composition of the non-recirculating Krebs–Henseleit perfusate was as follows (mM): NaCl 118, KCI 4.7, CaCI_2_ × 2H_2_O 2.5, MgSO_4_ × 7H_2_O 1.7, NaHCO_3_ 25, KH_2_PO_4_ 1.2, glucose 11, pyruvate 2, equilibrated with 95% O_2_ plus 5% CO_2_ and warmed to 37°C (pH 7.4). Immediately after the restoration of a normal heart rhythm, a sensor (transducer BS473-0184, Experimetria Ltd, Budapest, Hungary) was inserted into the left ventricle for continuous monitoring of cardiac function through a created entrance to the left atrium of the heart and damaged mitral valve.

### Physiological Assay and Experimental Protocol on Langendorff Apparatus

The hearts from all groups underwent a 20-min stabilization perfusion period at CPP of 70 cm H_2_O. CTRL underwent perfusion for 1 h and 30 min without any intervention. In the CTRL group, hearts were subjected to ischemic preconditioning lasting 5 min before global ischemia (perfusion was totally stopped) for 30 min followed by 1 h of reperfusion. Both rat exercise groups (ETr and HIITr) went through the same ischemia/reperfusion injury protocol 36 h after following the exercise protocol. After stabilization, the hearts were under global ischemia for 30 min followed by 1 h of reperfusion. Using the sensor within the left ventricle, the following parameters of myocardial function were determined:

1. Maximum rate of pressure development in the left ventricle (dp/dt max)
2. Minimum rate of pressure development in the left ventricle (dp/dt min)
3. Systolic left ventricular pressure (SLVP)
4. Diastolic left ventricular pressure (DLVP)
5. Heart rate (HR).

Coronary flow (CF) was measured flowmetrically and collected at the following intervals: in stabilization (S), in reperfusion at the first, third, fifth, and tenth minute, and 15-min intervals following the 10th minute (R1–R60).

### Markers of Oxidative Stress

The following oxidative stress parameters were determined spectrophotometrically (Specord S-600 Analytik Jena) using collected samples of the coronary venous effluent:

1. The index of lipid peroxidation, measured as thiobarbituric acid reactive substances (TBARS)
2. The level of nitrite (${\text{NO}}_{2}^{\;-}$)
3. The level of the superoxide anion radical (${\text{O}}_{2}^{-}$), and
4. The level of hydrogen peroxide (H_2_O_2_).

#### Thiobarbituric Acid Reactive Substances Determination (Index of Lipid Peroxidation)

The degree of lipid peroxidation in the coronary venous effluent was estimated by measuring TBARS, using 1% thiobarbituric acid in 0.05 NaOH, which was incubated with the coronary effluent at 100°C for 15 min and measured at 530 nm. Krebs–Henseleit solution was used as a blank probe (Ohkawa et al., 1979).

#### Determination of the Nitrite Level

Nitric oxide decomposes rapidly to form stable nitrite/nitrate products. The nitrite level (${\text{NO}}_{2}^{-}$) was measured and used as an index of nitric oxide (NO) production, using Griess's reagent. A total of 0.5 ml of perfusate was precipitated with 200 μl of 30% sulfo-salicylic acid, vortexed for 30 min, and centrifuged at 3,000 × g. Equal volumes of the supernatant and Griess's reagent, containing 1% sulfanilamide in 5% phosphoric acid/0.1% naphthalene ethylenediamine-dihydrochloride were added and incubated for 10 min in the dark and measured at 543 nm. The nitrite levels were calculated using sodium nitrite as the standard (Green et al., 1982).

#### Determination of the Level of the Superoxide Anion Radical

The level of the superoxide anion radical (${\text{O}}_{2}^{-}$) was measured via a nitro blue tetrazolium (NBT) reaction in TRIS buffer with coronary venous effluent, at 530 nm. Krebs–Henseleit solution was used as a blank probe (Auclair and Voisin, 1985).

#### Determination of the Hydrogen Peroxide Level

The measurement of the level of hydrogen peroxide (H_2_O_2_) was based on the oxidation of phenol red by hydrogen peroxide in a reaction catalyzed by horseradish peroxidase (HRPO) (Pick and Keisari, 1980). Two hundred microliters of perfusate was precipitated using 800 ml of freshly prepared phenol red solution; 10 μl of (1:20) HRPO (made *ex tempore*) was subsequently added. For the blank probe, an adequate volume of Krebs–Henseleit solution was used instead of coronary venous effluent. The level of H_2_O_2_ was measured at 610 nm.

### Assessment of Blood Lactate Concentration

Levels of blood lactate were determined using a portable monitor (Lactate Plus, Nova Biomedical, Waltham, MA, USA) and lancet (Owen Mumford, Inc., Marietta, GA, USA). After 5 min of rest, blood was taken from the tail vein, and changes in lactate levels in response to training were calculated as the difference in the sum of lactate concentration after training vs. health control and group exposed to other exercise protocols (Astorino et al., 2019).

### Statistical Analysis

Values were expressed as the mean ± standard error. Before statistical analysis, all data were checked for normality using the one-sample Kolmogorov–Smirnov test. Biochemical data were evaluated using one-way analysis of variance (ANOVA and the *post-hoc* Bonferroni test for multiple comparisons). IBM SPSS Statistics 22.0 for Windows was used for statistical analysis of data. By statistical analysis, the results in the group itself were compared especially referring to three points of interest: stabilization point (S), first minute of reperfusion (R1), and last minute of reperfusion (R60). Values of *p* < 0.05 were considered to be statistically significant, while values of *p* < 0.01 were considered to be highly statistically significant.

## Results

### The Effects of High-Intensity Interval Training and Endurance Training on the Cardiodynamic Parameters of Isolated Rat Heart

#### Maximum Rate of Pressure Development in Left Ventricle (dp/dt max)

**Figure 1:**
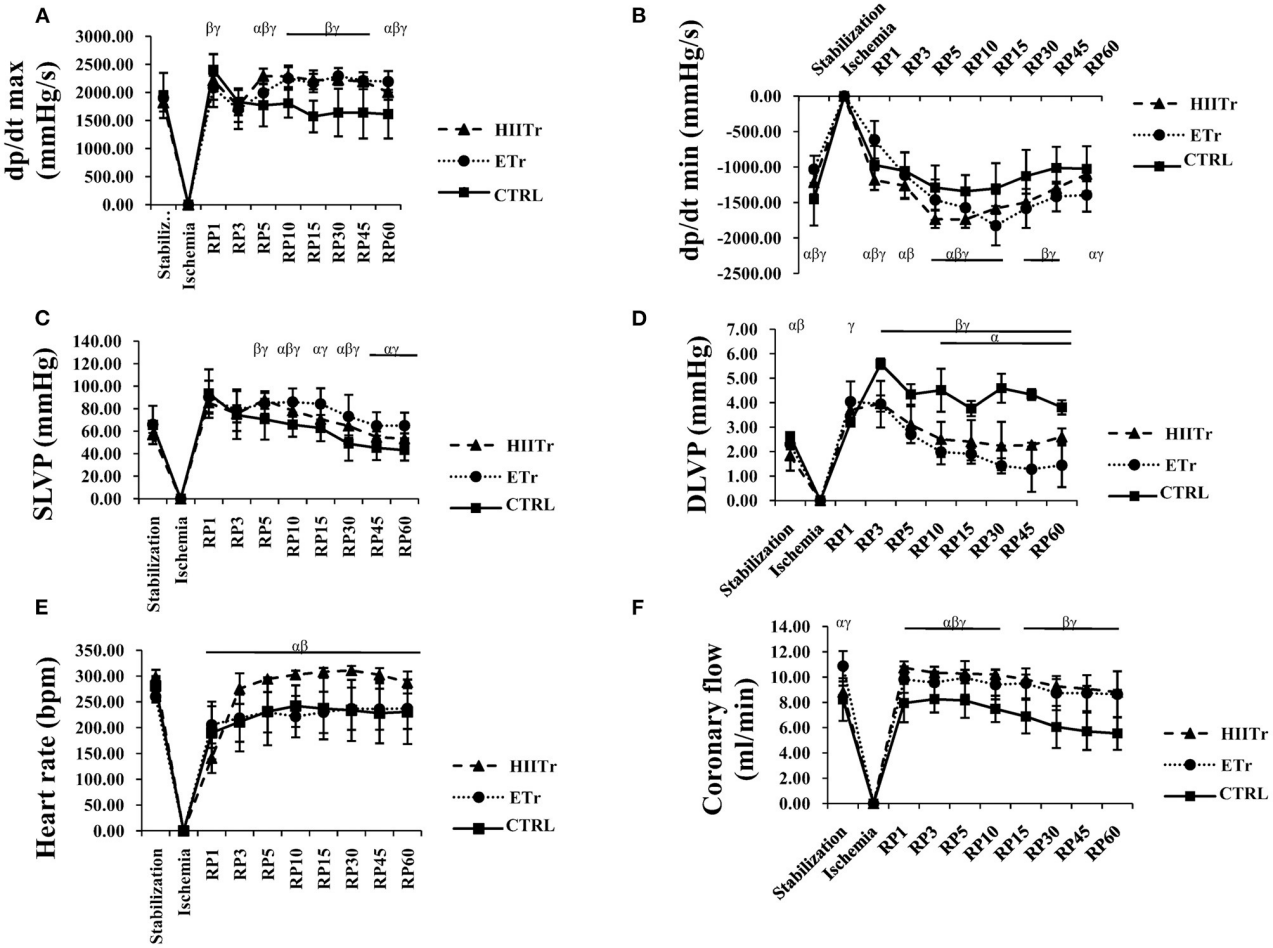
Effects of high intensity interval training (HIITr) and endurance training (ETr) on the value of maximum rate of pressure development in the left ventricle (dp/dt max) **(A)**, minimum rate of pressure development in the left ventricle (dp/dt min) **(B)**, systolic left ventricular pressure (SLVP) **(C)**, diastolic left ventricular pressure (DLVP) **(D)**, heart rate **(E)**, coronary flow **(F)**. α statistical significance at the level of *p* < 0.05 between HIITr and ETr groups; β statistical significance at the level of *p* < 0.05 between HIITr and sedentary control (CTRL) groups; γ statistical significance at the level of *p* < 0.05 between ETr and CTRL groups; Data are presented as means ± SD and analyzed through points of interest: stabilization (S), first minute of reperfusion (R1), last minute of reperfusion (R60).

In the control group, there was a significant increase in dp/dt max in the first point of reperfusion (R1) in comparison to the stabilization period and last moment of reperfusion (R60). The value of this parameter in R1 was similar to the stabilization period (S) in the HIITr and ETr groups, but the hearts from the HIITr group showed a better response in the other point of reperfusion period (after ischemia) and less fluctuation in this cardiodynamic parameter compared to those in the ETr group (Figure 1).

#### Minimum Rate of Pressure Development in Left Ventricle (dp/dt min)

The most prominent changes of dp/dt min were observed at the end of reperfusion (R60) in comparison to the stabilization (S) and first point of reperfusion (R1) in the ETr group, while in the control group at the last point of the recovery period (R60), dp/dt min value was higher compared to the values before ischemia (S). On the other hand, the values of this parameter in the HIITr group were similar at the first and last points of reperfusion to the period of stabilization (Figure 1).

#### Systolic Left Ventricular Pressure (SLVP)

At the end of the recovery period (R60) in the control group, the value of SLVP was significantly decreased in comparison to the values in stabilization (S). Also, a rise in values of SLVP was noticed at the first point of reperfusion (R1) compared to the stabilization point (S) in the HIITr and ETr groups, while at the end of the 60-min reperfusion, the value of SLVP in the HIIT group returned to the value detected before ischemia, which was not noticed in the ETr group where the value of SLVP remained increased after the period of reperfusion (Figure 1).

#### Diastolic Left Ventricular Pressure (DLVP)

A significant increase in the DLVP values in all groups during the period of reperfusion was noticed compared with the stabilization period. Furthermore, at the end of the observed period (R60), the DLVP values in all groups did not return to the value before ischemia (Figure 1).

#### Heart Rate (HR)

The most significant drop in HR was observed in the HIITr group at the first point of reperfusion (R1) when compared with the stabilization period (S). However, in the remaining periods of reperfusion, the value of HR in this group stayed constant and returned to the same value before ischemia. In the other two groups, control, and ETr, HR values were significantly decreased at the end of the recovery period (R60) in comparison with the values in stabilization (S) (Figure 1).

#### Coronary Flow (CF)

In the control group, the CF value was the same at the first point of reperfusion (R1) compared with the stabilization period (S), while there was a prominent decrease at the last point of reperfusion (R60) when compared with the stabilization period (S). Additionally, in the HIITr group, a rise in CF during the observed points of interest was noticed, while in the ETr group, CF was remarkably decreased in the first point of reperfusion (R1) as well as other points of the observed period (Figure 1).

### The Effects of High-Intensity Intensive Training and Endurance Training on Markers of Oxidative Stress

#### Index of Lipid Peroxidation (Measured as Thiobarbituric Acid Reactive Substances)

The index of lipid peroxidation in coronary venous effluent did not differ significantly in the control and HIITr group, while in the ETr group, there was a rise in the first minute of reperfusion (R1) compared with the value at the end of the recovery period (R60) (Table 3). Values of TBARS were higher in the CTRL and HIITr groups in comparison with ETr in all points of interest, while there were no statistical differences in levels of this prooxidant marker between the HIITr and CTRL groups.

**Table 3:** Oxidative stress parameters from coronary venous effluent within and between groups.

**Groups/Parametars**	**TBARS**	**${\text{O}}_{2}^{-}$**	**NO**	**H_**2**_O_**2**_**
**CTRL**				
Stabilization (S)	15.33 ± 2.90^a^	28.75 ± 2.51^aa^^,^ ^bb^	100.27 ± 12.19^a^^,^ ^b^	26.36 ± 2.81^a^
First minute of reperfusion (R1)	17.31 ± 1.98^a^	58.42 ± 3.09^aa^^,^ ^bb^	100.09 ± 17.76^aa^^,^ ^bb^	22.66 ± 1.37^a^^,^ ^b^
1 h of reperfusion (R60)	13.54 ± 1.54^a^	27.66 ± 2.69^aa^^,^ ^b^	61.47 ± 9.19^aa^^,^ ^bb^	19.30 ± 1.73^a^^,^ ^b^
S vs. R1 (%)	12.93	103.23*	−0.18	−14.03
S vs. R60 (%)	−11.70	−3.76	−38.69**	−26.79*
R1 vs. R60 (%)	−21.81	−52.65*	−38.58**	−14.84
**ETr**				
Stabilization (S)	11.09 ± 1.72^a^^,^ ^c^	43.79 ± 10.86^aa^^,^ ^cc^	111.88 ± 14.38^a^^,^ ^c^	38.88 ± 1.87^a^^,^ ^c^
First minute of reperfusion (R1)	12.71 ± 1.38^a^^,^ ^c^	76.53 ± 9.70^aa^^,^ ^cc^	127.16 ± 1.88^aa^	34.64 ± 1.99^a^^,^ ^c^
1 h of reperfusion (R60)	8.23 ± 0.73^a^^,^ ^c^	241.79 ± 10.16^aa^^,^ ^cc^	95.55 ± 5.57^aa^^,^ ^c^	25.48 ± 1.76^a^
S vs. R1 (%)	14.64	74.76	13.65	−10.92*
S vs. R60 (%)	−25.79	452.14*	−14.60	−34.48*
R1 vs. R60 (%)	−35.26**	215.95*	−24.86**	−26.45*
**HIITr**				
Stabilization (S)	14.04 ± 1.06^c^	89.54 ± 5.27^bb^^,^ ^cc^	112.27 ± 2.41^b^^,^ ^c^	26.83 ± 1.02^c^
First minute of reperfusion (R1)	15.83 ± 1.84^c^	34.14 ± 3.05^bb^^,^ ^cc^	125.42 ± 3.80^bb^	28.78 ± 1.17^b^^,^ ^c^
1 h of reperfusion (R60)	15.56 ± 1.10^c^	36.90 ± 2.79^b^^,^ ^cc^	112.30 ± 0.54^bb^^,^ ^c^	26.60 ± 2.48^b^
S vs. R1 (%)	12.79	−61.87	11.71	7.27
S vs. R60 (%)	10.86	−58.78**	0.03	−0.85
R1 vs. R60 (%)	−1.71	8.11	−10.46	−7.57

Mean values ± SD in three points of interest. Percentage increase or decrease between points of interest within groups with statistical difference (p < 0.05*, p < 0.01**).

a Statistical significance at the level of p < 0.05 between endurance training (ETr) and sedentary control (CTRL) groups;

aa statistical significance at the level of p < 0.01 between ETr and CTRL groups;

b statistical significance at the level of p < 0.05 between HIITr and CTRL groups;

bb statistical significance at the level of p < 0.01 between HIITr and CTRL groups;

c statistical significance at the level of p < 0.05 between HIITr and ETr groups;

cc *statistical significance at the level of p < 0.01 between HIITr and ETr groups*.

Bold values represented significant differences within the group, while * represents high statistical significance (p < 0.05) and ** represents very high statistical significance (p < 0.01).

#### Level of Nitrites (${\text{NO}}_{2}^{\text{–}}$)

There was a drop in ${\text{NO}}_{2}^{-}$ production in the control group at the last point of recovery period (R60) in comparison with the first period of reperfusion (R1) and stabilization (S). Furthermore, in the ETr group, a higher value of ${\text{NO}}_{2}^{-}$ was noticed in the first minute of reperfusion (R1) when compared with the end of this period (R60). In the HIITr group, the level of ${\text{NO}}_{2}^{-}$ did not vary significantly (Table 3). Notably, higher ${\text{NO}}_{2}^{-}$ values were detected in the HIITr and ETr groups compared with the CTRL during the observed periods, while the levels of this marker did not differ significantly between the two experimental groups.

#### Level of Superoxide Anion Radical (${\text{O}}_{2}^{\text{–}}$)

The level of ${\text{O}}_{2}^{-}$ in the control group was significantly increased in the first period of recovery (R1) in comparison with the level before ischemia (S) and at the end of the reperfusion period (R60). However, a rise in the value of ${\text{O}}_{2}^{-}$ in the ETr group was noticed at the end of the observed period (R60) compared with stabilization (S) and the first minute of reperfusion (R1). In the HIITr group, a difference in the level of ${\text{O}}_{2}^{-}$ during the period of interest was also observed, especially at the period of stabilization (S) in comparison with the end of the period of recovery (R60) (Table 3). A pronounced rise in the level of this oxidative stress marker was detected in groups exposed to exercise protocols than in the control group, except the R1 moment when the value of ${\text{O}}_{2}^{-}$ was higher in CTRL compared with the HIITr group. Furthermore, a significantly greater level of this marker was observed in the HIITr group at the stabilization period compared with ETr, until the period of reperfusion when the ${\text{O}}_{2}^{-}$ level was decreased in the HIITr in comparison with the ETr group.

#### Level of Hydrogen Peroxide (H_2_O_2_)

In the HIITr group, the level of H_2_O_2_ did not differ significantly, while an increase in H_2_O_2_ at the stabilization period (S) in control group was noticed in comparison with the end of reperfusion period (R60). Interestingly, in the ETr group, it was a remarked significant difference in the H_2_O_2_ level among all points of interest. At the stabilization period (S), H_2_O_2_ was increased compared with the first (R1) and the last period of recovery (R60), and, also there was a significant increase in H_2_O_2_ at the first period of reperfusion (R1) in comparison with the end of the observed period (R60) (Table 3). Additionally, the level of this marker was lower in the control group compared with the other two groups in all the experimental moments, except the stabilization period when we observed the same trend of H_2_O_2_ between the CTRL and HIITr groups. Comparing the impact of the different exercise regimens on the H_2_O_2_ value, we detected higher levels of this marker in the ETr group compared with HIITr except the last point of reperfusion when we did not observe any differences in H_2_O_2_ value between these two groups.

### Lactate Levels

After HIIT, the level of lactate was significantly increased compared with the health control group (*p* < 0.01) as well as in comparison with ETr (*p* < 0.05). Moreover, a higher lactate value was observed in the group exposed to physical activity of moderate intensity in comparison with the control group (*p* < 0.05). Values of lactate levels are demonstrated in Table 4.

**Table 4:** Lactate levels for analyzed groups of rat (Mean values ± SD).

**Groups/Parameters**	**Lactate levels (mM/L)**
CTRL	2.5 ± 0.4
ETr	3.5 ± 0.6
HIITr	4.6 ± 0.7

## Discussion

Physical activity such as exercise training is commonly regarded as an effective intervention to improve cardiovascular function, and the health benefits of regular physical activity are certainly irrefutable. It has been proved that exercise training is useful to increase cardiorespiratory fitness, a strong indicator of good metabolic health, low morbidity, and low risk of death (Fiuza-Luces et al., 2018; Feng et al., 2019). However, a great health concern has been focused on inappropriate exercise, which might lead to unexpected death especially for CVD individuals (Cobb and Weaver, 1986). In that sense, animal exercise models have been considered a powerful tool for assessing the impact of various exercise procedures, which would be a basis for designing individualized exercise programs in humans.

In the present study, through different experiments, we demonstrated numerous positive and beneficial effects of exercise in general. However, our main goal was to show that cardioprotection, via cardiac improvements, can occur with only 15 min of high intensity exercise. Besides, the fact that HIITr has gained great scientific attention, underlying mechanisms for its cardioprotective abilities, still remains unclear. To examine the effects of exercise intensity in providing cardioprotection in an ischemic insult, we created I/R on a rat heart model *in vitro* after endurance training (ETr) and high intensity interval training (HIITr). Using the *Langendorff* apparatus, we collected cardiodynamic parameters and coronary flow.

The main finding of the present study was that hearts from the HIITr group showed a better response in the reperfusion period (after ischemia) and less fluctuation in almost all observed cardiodynamic parameters, specifically regarding heart rate and coronary flow (Figure 1). Our data are in agreement with previous studies, which showed that short-term HIITr protects the heart against an *in vivo* I/R injury for at least 7 days following detraining, while there were no statistically significant differences on values of heart rate and mean arterial blood pressure among the experimental group (Rahimi et al., 2015; Kelly et al., 2018). Regarding long-term physical activity, Bowles et al. reported that coronary flow during early reperfusion after global ischemia in the isolated rat heart was greatest in animals that had high-intensity treadmill running compared with a lower intensity regimen (Bowles et al., 1992). This is in line with our finding showing that the value of coronary flow in the HIITr group was in considerable increment in the first point of recovery period (R1) in contrast to the two other groups (Figure 1). The fact that the value of this parameter returned to baseline values at the end of reperfusion (R60) suggests that preconditioning with short-term training preserved coronary vasodilatory response to I/R. On the other hand, Brown et al. pointed out that there were no differences in baseline coronary flow between groups, while after the onset of ischemia, coronary flow, in groups in which hearts underwent short-term training, and the control group, decreased similarly. However, after 45 min of ischemia, coronary flow was significantly higher in experimental than in the control group, and this difference persisted for the duration of the protocol (*p* < 0.05) (Brown et al., 2003). To evaluate myocardial contractility, we used dp/dt max (Figure 1) as an indirect indicator of inotropic properties of the heart, while dp/dt min (Figure 1) was measured as the rate of relaxation of the heart. Our results regarding dp/dt max show that after both types of training, the value of this parameter in the first point of reperfusion (R1) was similar to that of the stabilization period, but the hearts from the HIITr group showed better response in the other point of reperfusion period compared with those in the ETr group. Relaxing parameter of the myocardium is in accordance with inotropic characteristics, pointing out that HIITr, decreasing the power of contraction, diminishes the relaxation of the myocardium during all periods of recovery. Brown and co-workers found that high-intensity interval exercise does not affect the value of the left ventricule pressure, until 30 min into the reperfusion period when a significant increase in pressure is noticed, compared with the hearts from the control group (Brown et al., 2003). On the contrary, in the present study, it registered an increase of SLVP in the HIITr group already after the first minute of reperfusion, while at the end of the 60-min reperfusion, the value of SLVP returned to those detected before ischemia (Figure 1). Moreover, a rise in DLVP value after both types of exercise protocol during all times of observed period (Figure 1) was noticed, which is again is contrary to a previous study (Brown et al., 2003) where a lower diastolic pressure from 45 min of ischemia through 30 min of reperfusion was detected.

In a sedentary group where we applied just I/R protocol, a low and slow response from hearts during reperfusion was noticed. Values at the end of the reperfusion period (R60) were significantly different from those at the beginning (S), especially regarding coronary flow, systolic left ventricular pressure, and dp/dt min. In relation to the percentage change between the points of interest, the hearts from the ETr group had less fluctuation in the examined parameters compared with those of the control group, while the HIITr group incurred even less change than ETr.

According to the literature, it has been observed that inotropic properties of exercise activity are based on two different mechanisms. One way is related to favoring mitochondrial function and ATP production, while another mechanism is associated with a more effective function of the sarcoplasmatic reticulum in the sense of Ca^2+^ release and return into the sarcoplasmatic reticulum. In this way, muscle strength is stronger, and fatigue is reduced (Mahjoub et al., 2019). However, the mechanisms involved in the superiority of HIIT over ET are not clearly elucidated. One of the reasons for the aerobic capacity improvement during HIIT can be clarified through intracellular signaling sequence. Considering that HIIT induced muscular stimulus, AMPK activity in muscle cells increased leading to higher PGC-1α mRNA, protein level, and mRNA of the mitochondrial oxygenation enzyme (Ito et al., 2016). It was suggested that exercise might activate signaling pathways related to mitochondrial biogenesis, which involve phosphorylation of 5′ AMP-activated protein kinase and p38 MAPK, and the increased expression of PGC-1α mRNA. It was also shown that high expression PGC-1α through miR-322 induces improved mitochondrial function (Jeremic et al., 2020).

To evaluate the exercise training-induced cardiac growth, the heart weight/body mass ratio of the rats was assessed in the present study. We observed increases in the heart weight to bodyweight ratio in ETr and HIITr compared with that of the control (Table 5), however, without statistical significance. Moreover, a reduction in body weight after both types of exercise protocol was noticed. Previous research (Ghahremani et al., 2018) has also shown a higher heart weight/body mass ratio in the group that underwent a high-intensity interval training, exercising aerobically on a treadmill for 8 weeks and then applying the I/R protocol at the end of the training period, thus proving the effectiveness of the aerobic exercise training protocol on the cardiac muscle.

**Table 5:** Body weight, heart weight, and heart weight/body weight ratio for analyzed groups of rat.

**Groups/Parameters**	**BW before (g)**	**BW after(g)**	**ΔBW (g)**	**HW (g)**	**HW/BW ratio**
**CTRL**	266.05 ± 5.5	277.07 ± 3.75^*^^#^	11.02	1.13 ± 0.03	0.0041
**ETr**	258.66 ± 3.11	251.18 ± 3.87^*^	7.48	1.16 ± 0.05	0.0046
**HIITr**	259.31 ± 4.02	252.85 ± 4.11^#^	6.46	1.24 ± 0.07	0.0049

Values are presented as mean values ± SD (p < 0.05*^#^, p < 0.01**^##^).

* Statistical significance between ETr and CTRL groups;

# *statistical significance between HIITr and CTRL groups*.

Physical exercise has many benefits, but it might also have a negative influence on the body, commonly attributed to an imbalance between the levels of antioxidants and reactive oxygen and nitrogen species due to excessive production of free radicals during physical exercise (Bloomer et al., 2005; Finkler et al., 2014). While the most important variable in determining cardioprotection is the exercise intensity (Rankin et al., 2012), a significant focus should also be oriented to the duration of exercise (Kavazis, 2009). Consequently, as oxidative tissue status can be changed as a result of high intensity or moderate duration exercise, we tested oxidative stress markers in coronary venous effluent. Our findings indicate that the HIITr group showed less release of ROS in the reperfusion period than when we observed the ETr and CTRL (Table 3). In both the ETr and CTRL groups, we noticed a greater fluctuation in the release of the superoxide anion radical (${\text{O}}_{2}^{-}$) and hydrogen peroxide (H_2_${\text{O}}_{2}^{-}$); however, in HIITr, those changes were minimal. Similar results were observed in the study of *Stanojevic D*. and co-workers where the levels of ${\text{O}}_{2}^{-}$ and H_2_O_2_ were lower in the hearts from both moderately trained and overtrained rats (Stanojevic et al., 2016). Furthermore, superoxide, as a free radical, has a relatively long half-life, and it is primarily formed as an intermediate in biochemical reactions. Although superoxide anion radical is considered as relatively unreactive compared with the other radical species, this molecule can rapidly react with NO forming unusual peroxide, peroxynitrite. Consequently, the concentrations of these molecules in the experimental groups are increased probably because of the possible interaction of ${\text{O}}_{2}^{-}$ with NO^−^, consequently leading to peroxynitrite formation (Radi, 2018). On the other hand, appropriate levels of antioxidant enzymes are of sustainable importance due to their role to inhibit the formation of ROS. Superoxide dismutase prevents the accumulation of ${\text{O}}_{2}^{-}$ by accelerating its dismutation to H_2_O_2_ (Steinbacher and Eckl, 2015). We have found that training induced slightly higher values of H_2_O_2_ compared with the control group. These results can be explained by the accepted idea that higher H_2_O_2_ steady state levels are observed due to overexpression of CuZn-SOD (Teixeira et al., 1998). Through this report, we can conclude that physical activity induces greater antioxidant defense, which is reflected in a higher level of H_2_O_2_ as a consequence of neutralization of O_2_ by SOD. Measuring TBARS is one of the ways to assess the degree of lipid peroxidation, which is one of the adverse redox change consequences. In our study, the value of this pro-oxidative parameter did not differ significantly in the HIITr group, while it a greater fluctuation in the release of TBARS in the ETr group during reperfusion period was noticed. Furthermore, in the HIITr group, lower levels of TBARS were observed in the ETr, which points to the better redox state after short-term training protocol. An additional factor that may contribute to at least some validation of training protocols in terms of aerobic/anaerobic quantification is metabolic stress. Although there are different methods to monitor this factor, one of the possible ways is via measurement of blood lactate concentration. Studies have shown that this parameter may be an index of intramuscular stress and is associated with muscle fatigue and decreased malleability as the response to the training (Spriet et al., 2000; Astorino et al., 2019). Our data showed higher blood lactate level in the HIITr group compared with those in the other groups. Such results arise as a consequence of HIIT due to lack of time for lactate metabolic degradation. Additionally, lactate levels in the ETr group were also increased compared with those in the CTRL group but significantly lower than those in the HIITr group due to lower intensity of physical activity and longer periods of resting between training sessions. These results are in line with previously published data where they showed increased lactate level in HIIT compared with long-distance training concluding that the HIIT regime is more superior for lactate production (Ní Chéilleachair et al., 2017).

In the end, it is important to emphasize that all study findings must be observed in light of the fact that the Langendorff model does not take into account the heart rate variability (HRV) and vagus nerve influence as the left anterior descending artery (LAD) ligation model. Another limitation of the study is the absence of results regarding the systemic effects of the two training regimes, which could certainly affect cardiac changes. In summary, although performed on an animal model, this study may help in the better understanding of the differences between the effects of HIITr/ETr training regimes on I/R injury. Therefore, it proves the beneficial impact of HIITr in cardioprotection. Also, our results can be of interest in explaining both exercise protocols' impact on redox status as well as for the possible clinical impact in the design of therapeutic strategies for cardioprotection. However, we were not able to investigate the possible physiological and molecular changes in both exercise protocols, which would be useful in highlighting unknown molecular mechanisms for cardioprotection. Accordingly, our future plans will be directed to reveal the possible expression and role of many molecules as well as the activation of the different pathways during both training regimes included in the reduction of cardiovascular risks.

## Conclusion

In the present study, we perceived the main functional differences between the two different styles of exercise that require very different time commitments, while both styles created beneficial cardiac adaptations. The main conclusion of this research is that preconditioning with five sessions of HIITr performed over a 5-week period may also be superior in protecting the heart against I/R injury. Given that a “lack of time” is the most often cited reason for not exercising regularly, this represents a more time-efficient training method for improving cardiac function than a more time-consuming ETr. Moreover, it seems that HIITr can encourage oxidative stress within the heart, which can be of interest for the elucidation of the impact of different exercise protocols on cardiac redox homeostasis.

## Data Availability Statement

The datasets generated for this study are available on request to the corresponding author.

## Ethics Statement

This study was carried out in accordance with the recommendations of European Directive for welfare of laboratory animals no: 2010/63/EU and Good Laboratory Practice (GLP) principles. The protocol for the current study was approved by the Ethics committee for experimental animal well-being of the Faculty Of Medical Sciences at the University of Kragujevac, Serbia (No: 01-13340/1).

## Author Contributions

MRan, VZ, IS, and MRav performed the experiments and collected the data. BJ and TT performed the statistical analyses. JB, IM, JJ, OM, and NJ performed the biochemical analyses and collected the biochemical data from the study. VJ and SB designed the study. All authors contributed to the interpretation of the results and the writing of the manuscript.

## Conflict of Interest

The authors declare that the research was conducted in the absence of any commercial or financial relationships that could be construed as a potential conflict of interest.

## References

[B1] Astorino T. A. DeRevere J. L. Anderson T. Kellogg E. Holstrom P. Ring S.. (2019). Blood lactate concentration is not related to the increase in cardiorespiratory fitness induced by high intensity interval training. Int. J. Environ. Res. Public Health 16:2845. 10.3390/ijerph16162845 31395812 PMC6720831

[B2] Auclair C. Voisin E. (1985). Nitroblue tetrazolium reduction, in Handbook of Methods for Oxygen Radical Research,. ed R. A. Greenvvald (Boca Raton, FL: CRC Press), 123–132.

[B3] Bloomer R. J. Goldfarb A. H. Wideman L. McKenzie M. J. Consitt L. A. (2005). Effects of acute aerobic and anaerobic exercise on blood markers of oxidative stress. J. Strength Cond. Res. 19, 276–285. 10.1519/14823.1 15903362

[B4] Boutcher S. H. (2011). High-intensity intermittent exercise and fat loss. J. Obes. 2011:868305. 10.1155/2011/868305 21113312 PMC2991639

[B5] Bouzid M. A. Filaire E. Matran R. Robin S. Fabre C. (2018). Lifelong voluntary exercise modulates age-related changes in oxidative stress. Int. J. Sports Med. 39, 21–28. 10.1055/s-0043-119882 29169189

[B6] Bowles D. K. Farrar R. P. Starnes J. W. (1992). Exercise training improves cardiac function after ischemia in the isolated, working rat heart. Am. J. Physiol. 263, H804–H809. 1415606 10.1152/ajpheart.1992.263.3.H804

[B7] Brown D. A. Jew K. N. Sparagna G. C. Musch T. I. Moore R. L. (2003). Exercise training preserves coronary flow and reduces infarct size after ischemia-reperfusion in rat heart. J. Appl. Physiol. 95, 2510–2518. 10.1152/japplphysiol.00487.2003 12937028

[B8] Calvert J. W. (2011). Cardioprotective effects of nitrite during exercise. Cardiovasc. Res. 89, 499–506. 10.1093/cvr/cvq307 20876585 PMC3028971

[B9] Cobb L. A. Weaver W. D. (1986). Exercise: a risk for sudden death in patients with coronary heart disease. J. Am. Coll. Cardiol. 7, 215–219. 3510234 10.1016/s0735-1097(86)80284-4

[B10] Costa E. C. Hay J. L. Kehler D. S. Boreskie K. F. Arora R. C. Umpierre D.. (2018). Effects of high-intensity interval training versus moderate-intensity continuous training on blood pressure in adults with pre- to established hypertension: a systematic review and meta-analysis of randomized trials. Sports Med. 48, 2127–2142. 10.1007/s40279-018-0944-y 29949110

[B11] de Lade C. G. Andreazzi A. E. Bolotari M. Costa V. M. G. Peters V. M. Guerra M. O. (2018). Effects of moderate intensity endurance training vs. high intensity interval training on weight gain, cardiorespiratory capacity, and metabolic profile in postnatal overfed rats. Diabetol. Metab. Syndr. 10:70. 10.1186/s13098-018-0374-x 30275910 PMC6158819

[B12] Feng R. Wang L. Li Z. Yang R. Liang Y. Sun Y.. (2019). A systematic comparison of exercise training protocols on animal models of cardiovascular capacity. Life Sci. 217, 128–140. 10.1016/j.lfs.2018.12.001 30517851 PMC6320317

[B13] Finkler M. Lichtenberg D. Pinchuk I. (2014). The relationship between oxidative stress and exercise. J. Basic Clin. Physiol. Pharmacol. 25, 1–11. 10.1515/jbcpp-2013-0082 23959662

[B14] Fiuza-Luces C. Santos-Lozano A. Joyner M. Carrera-Bastos P. Picazo O. Zugaza J. L.. (2018). Exercise benefits in cardiovascular disease: beyond attenuation of traditional risk factors. Nat. Rev. Cardiol. 15, 731–743. 10.1038/s41569-018-0065-1 30115967

[B15] Fletcher G. F. Ades P. A. Kligfield P. Arena R. Balady G. J. Bittner V. A.. (2013). American Heart Association Exercise, Cardiac Rehabilitation, and Prevention Committee of the Council on Clinical Cardiology, Council on Nutrition, Physical Activity and Metabolism, Council on Cardiovascular and Stroke Nursing, and Council on Epidemiology and Prevention. Exercise standards for testing and training: a scientific statement from the American Heart Association. Circulation 128, 873–934. 10.1161/CIR.0b013e31829b5b44 23877260

[B16] Frasier C. R. Moore R. L. Brown D. A. (2011). Exercise-induced cardiac preconditioning: how exercise protects your achy-breaky heart. J. Appl. Physiol. 111, 905–915. 10.1152/japplphysiol.00004.2011 21393468

[B17] Ghahremani R. Damirchi A. Salehi I. Komaki A. Esposito F. (2018). Mitochondrial dynamics as an underlying mechanism involved in aerobic exercise training-induced cardioprotection against ischemia-reperfusion injury. Life Sci. 213, 102–108. 10.1016/j.lfs.2018.10.035 30355530

[B18] Gibala M. J. Little J. P. Macdonald M. J. Hawley J. A. (2012). Physiological adaptations to low-volume, high intensity interval training in health and disease. J. Physiol. 590, 1077–1084. 10.1113/jphysiol.2011.224725 22289907 PMC3381816

[B19] Green D. J. Smith K. J. (2018). Effects of exercise on vascular function, structure, and health in humans. Cold Spring Harb. Perspect. Med. 8:a029819. 10.1101/cshperspect.a029819 28432115 PMC5880156

[B20] Green L. C. Wagner D. A. Glogowski J. Skipper P. L. Wishnok J. S. Tannenbaum S. R. (1982). Analysis of nitrate, nitrite, and [15N]nitrate in biological fluids. Anal. Biochem. 126, 131–138. 7181105 10.1016/0003-2697(82)90118-x

[B21] Hannan A. L. Hing W. Simas V. Climstein M. Coombes J. S. Jayasinghe R.. (2018). High-intensity interval training versus moderate-intensity continuous training within cardiac rehabilitation: a systematic review and meta-analysis. Open Access J Sports Med. 9, 1–17. 10.2147/OAJSM.S150596 29416382 PMC5790162

[B22] Ito S. Mizoguchi T. Saeki T. (2016). Review of high-intensity interval training in cardiac rehabilitation. Intern. Med. 55, 2329–2336. 10.2169/internalmedicine.55.6068 27580530

[B23] Jeremic N. Weber G. J. Theilen N. T. Tyagi S. C. (2020). Cardioprotective effects of high-intensity interval training are mediated through microRNA regulation of mitochondrial and oxidative stress pathways. J. Cell. Physiol. 235, 5229–5240. 10.1002/jcp.29409 31823395

[B24] Kavazis A. N. (2009). Exercise preconditioning of the myocardium. Sports Med. 39, 923–935. 10.2165/11317870-000000000-00000 19827860

[B25] Kelly D. T. Tobin C. Egan B. McCarren A. O'Connor P. L. McCaffrey N.. (2018). Comparison of sprint interval and endurance training in team sport athletes. J. Strength Cond. Res. 32, 3051–1058. 10.1519/JSC.0000000000002374 29373432

[B26] Kessler H. S. Sisson S. B. Short K. R. (2012). The potential for high-intensity interval training to reduce cardiometabolic disease risk. Sports Med. 42, 489–509. 10.2165/11630910-000000000-00000 22587821

[B27] Li Y. Cai M. Cao L. Qin X. Zheng T. Xu X.. (2014). Endurance exercise accelerates myocardial tissue oxygenation recovery and reduces ischemia reperfusion injury in mice. PLoS ONE 9:e114205. 10.1371/journal.pone.0114205 25474642 PMC4256403

[B28] Mahjoub H. Le Blanc O. Paquette M. Imhoff S. Labrecque L. Drapeau A.. (2019). Cardiac remodeling after six weeks of high-intensity interval training to exhaustion in endurance-trained men. Am. J. Physiol. Heart Circ. Physiol. 317, H685–H694. 10.1152/ajpheart.00196.2019 31347913

[B29] Meijers W. C. de Boer R. A. (2017). Exercise and heart failure: Improve your functional status and your biomarker profile. Eur. J. Prev. Cardiol. 24, 1358–1359. 10.1177/2047487317714849 28631932

[B30] Ní Chéilleachair N. J. Harrison A. J. Warrington G. D. (2017). HIIT enhances endurance performance and aerobic characteristics more than high-volume training in trained rowers. J. Sports Sci. 35, 1052–1058. 10.1080/02640414.2016.1209539 27438378

[B31] Ohkawa H. Ohishi N. Yagi K. (1979). Assay for lipid peroxides in animal tissues by thiobarbituric acid reaction. Anal. Biochem. 95, 351–358. 36810 10.1016/0003-2697(79)90738-3

[B32] Pick E. Keisari Y. (1980). A simple colorimetric method for the measurement of hydrogen peroxide produced by cells in culture. J. Immunol. Methods 38, 161–170. 6778929 10.1016/0022-1759(80)90340-3

[B33] Pinckard K. Baskin K. K. Stanford K. I. (2019). Effects of exercise to improve cardiovascular health. Front. Cardiovasc. Med. 4:69. 10.3389/fcvm.2019.00069 31214598 PMC6557987

[B34] Radi R. (2018). Oxygen radicals, nitric oxide, and peroxynitrite: redox pathways in molecular medicine. Proc. Natl. Acad. Sci. U.S.A. 115, 5839–5848. 10.1073/pnas.1804932115 29802228 PMC6003358

[B35] Rahimi M. Shekarforoush S. Asgari A. R. Khoshbaten A. Rajabi H. Bazgir B.. (2015). The effect of high intensity interval training on cardioprotection against ischemia-reperfusion injury in wistar rats. EXCLI J. 14, 237–246. 10.17179/excli2014-587 26417361 PMC4555214

[B36] Rankin A. J. Rankin A. C. MacIntyre P. Hillis W. S. (2012). Walk or run? Is high-intensity exercise more effective than moderate-intensity exercise at reducing cardiovascular risk? Scott. Med. J. 57, 99–102. 10.1258/smj.2011.011284 22194404

[B37] Rivas-Estany E. Sixto-Fernández S. Barrera-Sarduy J. Hernández-García S. González-Guerra R. Stusser-Beltranena R. (2013). Effects of long-term exercise training on left ventricular function and remodeling in patients with anterior wall myocardial infarction. Arch. Cardiol. Mex. 83, 167–173. 10.1016/j.acmx.2013.04.014 23906745

[B38] Serrano-Ostáriz E. Terreros-Blanco J. L. Legaz-Arrese A. George K. Shave R. Bocos-Terraz P.. (2011). The impact of exercise duration and intensity on the release of cardiac biomarkers. Scand. J. Med. Sci. Sports. 21,244–249. 10.1111/j.1600-0838.2009.01042.x 19919634

[B39] Simioni C. Zauli G. Martelli A. M. Vitale M. Sacchetti G. Gonelli A.. (2018). Oxidative stress: role of physical exercise and antioxidant nutraceuticals in adulthood and aging. Oncotarget 9, 17181–17198. 10.18632/oncotarget.24729 29682215 PMC5908316

[B40] Spriet L. L. Howlett R. A. Heigenhauser G. J. (2000). An enzymatic approach to lactate production in human skeletal muscle during exercise. Med. Sci. Sports Exerc. 32, 756–763. 10.1097/00005768-200004000-00007 10776894

[B41] Stanojevic D. Jakovljevic V. Barudzic N. Zivkovic V. Srejovic I. Parezanovic Ilic K.. (2016). Overtraining does not induce oxidative stress and inflammation in blood and heart of rats. Physiol. Res. 65, 81–90. 10.33549/physiolres.933058 26596327

[B42] Steinbacher P. Eckl P. (2015). Impact of oxidative stress on exercising skeletal muscle. Biomolecules 5, 356–377. 10.3390/biom5020356 25866921 PMC4496677

[B43] Teixeira H. D. Schumacher R. I. Meneghini R. (1998). Lower intracellular hydrogen peroxide levels in cells overexpressing CuZn-superoxide dismutase. Proc. Natl. Acad. Sci. U.S.A. 95, 7872–7875. 10.1073/pnas.95.14.7872 9653107 PMC20896

[B44] Xie B. Yan X. Cai X. Li J. (2017). Effects of high-intensity interval training on aerobic capacity in cardiac patients: a systematic review with meta-analysis. Biomed Res. Int. 2017:5420840. 10.1155/2017/5420840 28386556 PMC5366197

